# The Streptococcus Mucosus Capsulatus and Its Relation to Otology

**Published:** 1916-11

**Authors:** Homer A. Trotter

**Affiliations:** New York City


### The Streptococcus Mucosus Capsulatus and Its Relation to Otology.

By HOMER A. TROTTER, Ph. B., M. D.,

New York City.

It has been the practice in the Eno Laboratory of the New York Eye and Ear Infirmary, under the direction of Dr. George Dixon, to examine smears from every case of suppurative middle ear disease, acute or chronic, admitted to the wards for observation. At this institution they have abandoned the use of cultivations after having used them for a short time, as the germs present in the canal were found to be always mixed, and so much time is lost in separating them. As the mastoid pus in cases coming to operation was also examined and comparison showed that the predominating germ in the canal was found in the mastoid pus with sufficient frequency to make it a good rule to regard the predominating germ in the discharge from the middle ear responsible for the mastoid involvement, and I see no good reason to change the view. As a rule we find the predominating germ shown in the smear from canal pus, in pus from the middle ear, in practically pure culture in mastoid pus, in pus from brain abscess, spinal fluid in cases complicated by meningitis. Dr. Dixon says, ''During the past eleven years I have made 5496 examinations of aural pus. Of these 26.6 per cent has been mixed infection, that is to say that a number of mixed germs were present, and it is not advisable to charge the infection to any particular germ. The majority of these were chronic cases, and where a radical operation was done the mastoid smear usually showed mixed infection. Streptococcus occurred the predominating germ in 24.8 per cent; pneumoccus 12.2 per cent; staphlococcus (aureas and albus) 7.4 per cent; streptococcus mucosus capsulatus 4.8 per cent; spitillium of Vincent 1.8 per cent. The remaining 22.4 per cent is represented by miscellaneous, negative, etc., among which are placed the bacillus pyocyaneus, diptheria, tubercle, coli-communis, and Friedlander, etc.''

The streptococcus mucosus capsulatus, in the matter of relative virulence compared with these various micro organisms, our experience leads us to regard the streptococcus mucosus capsulatus as standing first, also first in the destruction of bone. Streptococcus pyogenes next, pneumococcus third, and staphlococcus fourth, in acute suppurative processes about the middle ear, the streptococcus mucosus capsulatus is the most insidiously destructive the otologist is called upon to combat, and this cannot be made emphatic enough. The streptococcus mucosus capsulatus in a manner stands alone as being most virulent. When streptococcus mucosus capsulatus is the infective germ in acute purulent middle ear disease will tend to recover equally with the other forms of serious infection, provided it is seen early and proper drainage established.

### THE ORGANISM.

### Streptococcus Mucosus Capsulatus.

Morphology: The organism occurs single, in pairs, and short chains, the chains contain about eight to ten pairs, it resembles the pneumonococcus, but is slightly larger and takes the capsular stain. It is gram positive and stains with the usual aniline dyes, but is best stained with dilute carbo-fuchsin. It is not motile and does not form spores. The capsule presists in artificial cultivation.

Cultures: It grows either in the presence or absence of oxygen, the organism dries out rather quickly in cultures. Media containing glucose the organisms life is longer. Blood serum (Loeffler's Mixture) the colonies appear as light colorless mucus, irregular in outline and become confluent to form large patches of mucus like material. The water condensation is slightly cloudy. Glucose agar-growth along the line of innoculation—slants—hemispherical masses of the growth extend into the surrounding media, in the vertical plane.

Gelatin—no growth (in room temperature).

Plain Agar Slants—a very slight growth.

Bouillion Plain—the growth is slight if at all.

Glucose Bouillion—a slight cloudiness with a small amount of stringy looking sediment in the bottom of the tube.

Glycerin Agar—similar to that of blood serum.

Potato—no growth.

Milk—is not coagulated.

Pathogensis: Eight white rats, peritoneal cavities innoculated—they all died within twelve to forty-eight hours. Five white rats innoculated at root of tail, all died in from twelve to forty-eight hours. Rabbits and guinea pigs experimented upon, all died after innoculation. Examination after death shows a widely extended oedema at the site of innoculation, the spleen is enlarged and the peritoneal cavity is filled with viscid like material.

Toxin: Bouillion cultures filtered (germ free) and the filtrate injected into five white rats with no results—therefore toxins are not formed.

Summary: The micro-organism is frequently mistaken for the pneumococcus. At times it will stain very well, and show the capsule distinctly, other times the capsule is difficult to make out, especially if over heated when fixing, and the stain is too old. (Dilute Carbo-Fuchsin).

CASE HISTORY

A male, seventeen years of age, in the service of Dr. Whiting, was admitted to the New York Eye and Ear Infirmary, on August 17th, 1916. He gave history of having had pain and constant headache for three weeks. Pain was dull in character and involved his left ear. Discharge from the left ear was evident, which begun three weeks previous, following bathing in the sea.

August 22nd, 1916: Incision made in left M. T., he was under observation and ran a temperature ranging from 100 degrees F. to 104.6 degrees F. until September 1st, 1916, (fourteen days). X-ray reports showed that a large area of pneumonatic bone on right side, with extensive zygomatic cells on both sides. Right side clear. Left side cloudy and appearance operative. Sinus not shown. Smear from canal —right—few staphalococcus and short streptococcus,—left—unsatisfactory. Blood count—reds 4,200,000—white—19,100 Differential—Polys—86.2—L.2.2.—small 11.6 — Eosinphiles — None—Masts—None—Mylocytes—None.

August 23rd, 1916: A diagnosis of O. M. P. A. Mastoiditis, left, was made, and a simple mastoid operation performed. Bone was found to be slightly necrosed. The antrum contained granulation tissue, but no free pus. In a few cells over the sinus free pus was found, and a smear taken showed a mixed infection, with streptococcus mucosus capsulatus predominating, small area over sinus was exposed. No dura exposed.

August 29th, 1916: Patient developed meningitis, his neck became stiff and drawn backward, Kernig's sign was present.

August 30th, 1916: Spinal fluid was withdrawn which came away under considerable pressure, cloudy, globulin, positive—Cell Counts—1180 M. M.

Culture from spinal fluid showed.

A pure growth of streptococcus mucosus capsulatus was obtained. Anti-streptococcus serum was used in treatment, but did not effect improvement.

Patient died after becoming irrational and greatly cyanosed.

Conclusion: That the streptococcus mucocus capsulatus is a variety of pneumococcus is held by some bacteriologists. Park and Williams, the Rockefeller Institute, and some others hold this view, and call this variety of pneumococcus, pneumococcus mucosus.

In 1905 Park and Williams placed this variety with the pneumococci. Newfeld, Cole and Dochez have shown that certain pneumococci may be grouped according to their agglutination reactions, and are as follows, viz.:

Group I. and II. Typical pneumococci.

Group III. Pneumoccus mucosus.

Group IV. Heterogeneous strains.

Dr. George Sloan Dixon and others are of the opinion that the microörganism under discourse is a streptococcus, and not a pneumococcus, because of its characteristic behavior on culture media, and the results of animal ennoculation.

From my work at the New York Eye and Ear Infirmary, and the Roosevelt Hospital Laboratory, I am inclined to believe with Dr. Dixon, however, either side are not definitely sure of its true position, and more work on this very destructive microörganism may establish its true identity.

---

**Disorders of the Thymus in Adults.** G. H. Hoxie, Kansas City, Med. Herald, Sept., gives the weight of the thymus as follows; at birth 230 grains; 2 years 437; 14 years 432; 25 years 353; 35 years 250; afterward 215., subject to individual variation. Removal in young animals causes stunting due to dystrophy of bones, changes occur in the spleen and adrenals and death usually occurs with cachexia. Hypertrophy usually produces a condition resembling hyasthenia gravis. Hyperplasia leads to both mechanic and chemic disturbance, thymic asthma and status lymphaticus so-called. Tumors are chiefly adenoma and teratoma, the former sometimes giving the picture of myasthenia, the latter the mechanic features of oppression and suffocation. Feeding experiments produce contradictory results. A persistent thymus gives a varied symptomatology but usually, there is weakness, shortness of breath and intestinal sluggishness. Diagnosis is made by percussions and X-rays.

---

**Strangulated Diaphragmatic Hernia.** Junior Vitac of Libourne, Jour. de Med. de Bordeaux, Aug., reports a case of intestinal obstruction with typic symptoms except that the patient could rise from the bed and, indeed, found partial relief from a crouching posture. Exploratory operation revealed a part of the ileum passing through the left diaphragm. It ruptured during withdrawal but the obstruction seemed to be relieved. Buried sutures were placed for an extent of 10 c.m., the peritoneum was flushed and drained. Death ensued the next morning. Necropsy showed that, in addition to the ileum, an intricate mass of transverse colon, loops of small intestine and epiploon were involved in the hernia. The hernia had undoubtedly existed for many years if it was not congenital.

